# Analysis of Perinatal Care in Zambia in the Context of WHO Recommendations

**DOI:** 10.3390/healthcare14162548

**Published:** 2026-08-14

**Authors:** Natasha Mussa, Małgorzata Nagórska

**Affiliations:** 1 Independent Researcher, Lusaka 10101, Zambia; 2 Faculty of Health Sciences and Psychology, Collegium Medicum, University of Rzeszów, 35-310 Rzeszów, Poland; 3 University Center for Research and Development in Health Sciences, University of Rzeszów, 35-310 Rzeszów, Poland

**Keywords:** perinatal care, antenatal care, intrapartum, postnatal care, Zambia

## Abstract

**Background/Objectives**: Perinatal care is essential for the health and well-being of women and adolescent girls from conception through the first year postpartum. The World Health Organization (WHO) provides evidence-based recommendations aimed at improving the quality of antenatal, intrapartum, and postnatal care. This study mapped 51 WHO perinatal care recommendations against Zambian guidelines to identify areas of alignment, as well as gaps and challenges related to their implementation. **Methods**: The review was conducted following Joanna Briggs Institute (JBI) guidance. Published recommendations and relevant documents issued from 2016 onwards were included. National guidelines, policy documents, professional standards, and literature related to perinatal care in Zambia were identified through searches of PubMed, Google Scholar, WHO resources, and relevant institutional websites. WHO recommendations were mapped against Zambian guidelines and classified according to their level of alignment. **Results**: The findings demonstrated significant alignment between WHO recommendations and Zambian guidelines in perinatal healthcare. However, important implementation gaps remain; for example, only 4.7% of pregnant women attended all eight recommended antenatal care visits. Intrapartum care largely aligns with WHO standards, though there are differences in pain-relief choices. Furthermore, difficulties in adopting WHO recommendations are mostly due to health system constraints. **Conclusions**: Most of the Zambian guidelines align with the WHO recommendations. However, ten were classified as partly aligned, and one did not align. This small proportion of non-alignment may reflect adaptation for a national context and require further evidence-based evaluation. In addition, consolidating existing recommendations, strengthening health-system capacity, and addressing implementation gaps may support the delivery of standardized, high-quality perinatal care and contribute to improved maternal and newborn health outcomes.

## 1. Introduction

This review adopts a broad maternal and newborn care continuum perspective, encompassing antenatal, intrapartum, and postnatal care from pregnancy through the first year postpartum. This period represents a major life transition for women and adolescent girls [1,2]. For a positive pregnancy experience and outcome, women must attain optimum perinatal care throughout this period. Perinatal care encompasses a broad range of evidence-based health interventions delivered throughout the antenatal, intrapartum, and postnatal periods [3,4,5,6,7].

Globally, the WHO provides comprehensive recommendations for perinatal healthcare that promote high-quality, effective, and equitable care. In line with the Sustainable Development Goals (SDGs), WHO envisions a world in which every pregnant woman and newborn receives quality care throughout pregnancy, childbirth, and the postnatal period [8,9].

However, in Sub-Saharan Africa, ensuring access to comprehensive perinatal care for pregnant women and adolescent girls remains a major public health concern. Key health indicators including maternal, neonatal, and stillbirth mortality rates, remain substantially higher than global targets and WHO expectations. Of the estimated 295,000 maternal deaths, a significant proportion of 66% occur in the region. Similarly, of an estimated 2.5 million neonatal deaths worldwide, approximately 41% occur in the region [10,11,12].

Like many countries in the region, Zambia has made significant progress; however, the global standards have not yet been achieved. According to the 2024 Zambia Demographic and Health Survey, the maternal mortality ratio was estimated at 187 per 100,000 live births. This estimated rate is significantly higher than the global SDG 3.1 target, which aims to reduce the maternal mortality ratio to less than 70 per 100,000 live births [13,14].

Perinatal care should be provided in accordance with WHO recommendations. This is important because improvements in maternal and newborn health contribute to global health outcomes. Furthermore, these recommendations provide evidence-based approaches that can be systematically adapted to the local context. This results in standardized perinatal care while maintaining their scientific integrity. Additionally, these recommendations are intended for use in Zambia. In Zambia, perinatal care is predominantly delivered through the publicly funded health system. This means that WHO recommendations are highly relevant to the Zambian context [4,15]. Despite this relevance, there is still limited evidence on the extent to which Zambian perinatal guidelines align with WHO recommendations. Previous studies have primarily focused on the utilization of perinatal healthcare services, including antenatal care coverage, hospital delivery, and postnatal coverage. To improve perinatal health outcomes, aligning Zambian guidelines with WHO recommendations helps identify gaps in perinatal health services and systems.

We aimed to systematically identify, synthesize, and assess all perinatal guidelines developed for use in Zambia by the Ministry of Health, other health authorities, and professional associations. Furthermore, we aimed to compare Zambia’s recommendations for perinatal care with the WHO recommendations for antenatal, intrapartum, and postnatal care to identify areas of divergence and potential challenges or gaps. The review addressed the following research question: To what extent do Zambian perinatal care recommendations align with WHO recommendations, and what implementation gaps and challenges exist?

## 2. Materials and Methods

### 2.1. Study Design

This review was conducted in accordance with the JBI methodology for evidence synthesis [16]. A PRISMA-style flow diagram was used to illustrate the process of document screening, eligibility assessment, and inclusion.

### 2.2. Framework for Defining Eligibility

**Figure 1:**
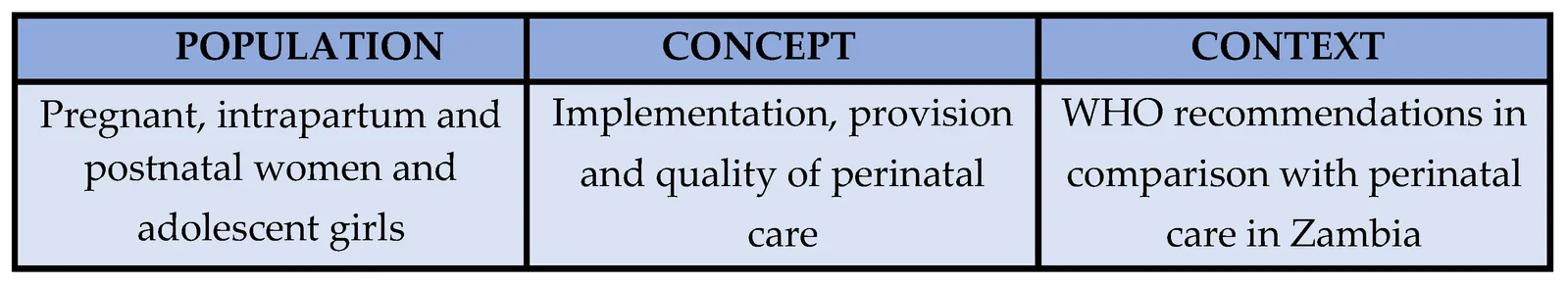
JBI Population-Concept-Context framework used in the review [16].

To define the eligibility criteria, the JBI Population-Concept-Context (PCC) frame-work was adopted, as shown in Figure 1 The population included pregnant women and adolescent girls, and women and adolescent girls during intrapartum and postnatal. The concept included the WHO recommendations for perinatal care, and the context of Zambia.

### 2.3. Eligibility and Inclusion Criteria

All documents published by the WHO, the Ministry of Health, Zambia, other health bodies in Zambia, and professional regulatory bodies intended for use as recommendations for antenatal, intrapartum, and postnatal care were included as eligible. Furthermore, documents that met one or more aspects of perinatal care were also included as eligible. These included assessments of antenatal care during pregnancy, intrapartum care during labour and childbirth, and postnatal care following birth. Because the definition of perinatal care may vary across sources, the search strategy included terms related to antenatal, intrapartum, and postnatal care. The search was restricted to documents published in English. The publication dates of the latest WHO recommendations were used to define the eligibility period; thus, only articles published in 2016 and onwards were included for the mapping analysis.

### 2.4. Exclusion Criteria

Exclusion criteria were established for recommendations intended for specific populations rather than the general population. Recommendations developed for high-risk pregnancies or for the management of specific medical conditions were excluded because the review focused on routine perinatal care applicable to the general population of pregnant women and newborns. Guidelines intended exclusively for use at the health facility level were also excluded. Policy documents that did not contain recommendations were excluded. Documents containing explicit recommendations were included only if they had been formally endorsed for use in Zambia.

### 2.5. Data Source, Search Strategy, and Screening

The data sources for the review included key reports of WHO recommendations on antenatal (2016) [9], intrapartum (2018) [17] and postnatal (2022) [18] mapped against Ministry Health Zambia reports and professional regulatory body guidelines. To obtain the data, a search for guidelines articles was conducted between 10th and 28th November 2025.

**Figure 2:**
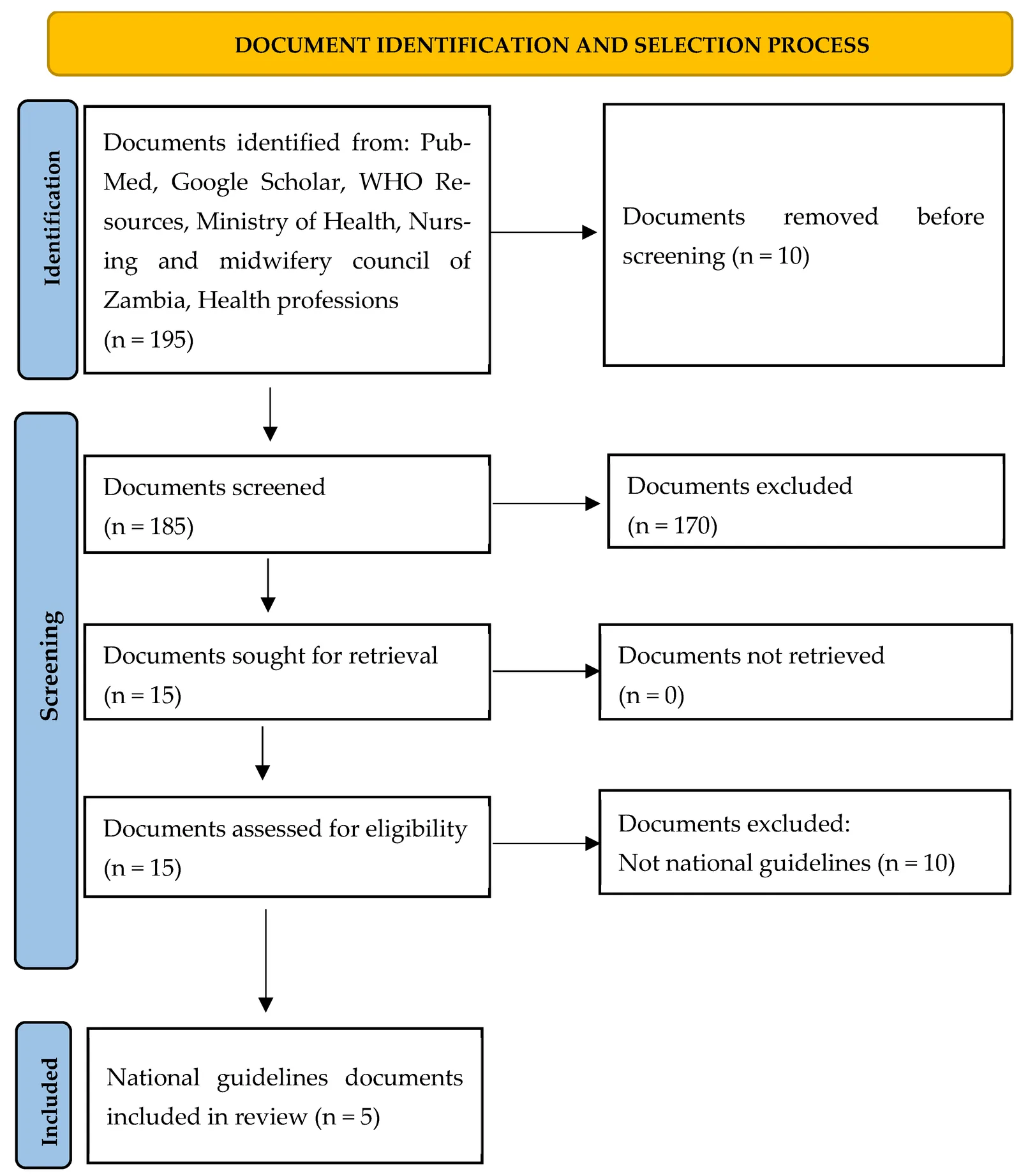
PRISMA-style flow diagram of the document selection process.

Additional searches were conducted on the websites of the Ministry of Health and relevant professional associations, such as the Nursing and Midwifery Council of Zambia (NMCZ) and the Health Professions Council of Zambia (HPCZ). Where official websites did not provide current information, supplementary Google searches were conducted to identify relevant policy documents and publications. Furthermore, the search items for titles and abstracts included “Perinatal Care in Zambia and Sub-Saharan,” “Maternal Health Care in Zambia,” “WHO recommendation,” and “Newborn Care in Zambia.” Identified records were screened for eligibility. The document selection process is presented using a PRISMA-style flow diagram (Figure 2).

Table 1 below presents the five national guidelines used in the mapping analysis. In addition, supplementary evidence such as the Zambia Demographic and Health. Survey and other relevant literature were cited in too provide contextual information on implementation on national coverage and understand the possible challenges that hinder the transitions of these guidelines into practice.

### 2.6. Data Extraction

A standardized Excel spreadsheet was developed to record the extracted data, including guideline characteristics (publisher, year, funding body, intended scope, and setting), and specific perinatal care recommendations. Data extraction focused on recommendations related to antenatal, intrapartum, and postnatal care. The classification framework used to assess the alignment between WHO recommendations and Zambian guidelines is presented in Table 2. The initial data extraction and recommendation mapping were conducted by the first author. The second author reviewed the extracted data and classifications, and any differences in interpretation or classification were discussed and resolved by consensus between the authors.

### 2.7. Recommendation Mapping

WHO recommendations served as the reference standard for comparison. Recommendations identified in Zambian guidelines were grouped according to the continuum of care (antenatal, intrapartum, and postnatal care) and mapped against the corresponding WHO recommendations. Each recommendation was classified according to its level of alignment with the WHO recommendation as described in Table 2. A total of 51 WHO recommendations relevant to routine perinatal care were included in the mapping process.

### 2.8. Data Analysis

In this review, a total of 51 WHO recommendations on perinatal care were analyzed. WHO recommendations were compared with Zambian guidelines using the classification framework presented in Table 2, and the detailed mapping. Narrative explanations were provided for the recommendation after mapping. Furthermore, potential reasons for discrepancies and implementation gaps were grouped into the following themes: shortage of healthcare professionals; limited access to equipment; inadequate maternal knowledge and health literacy; sociocultural practices; low antenatal care attendance and late initiation of antenatal care; weak continuity of postnatal care; broader health system; and limitations.

### 2.9. Interpretation and Synthesis

In the current study, the findings are presented in three categories. as follows: First, recommendations in Zambian guidelines that align with WHO recommendations. Second, recommendations that are only partially aligned are presented. Third, recommendations with major implementation gaps are identified and discussed.

## 3. Results

### 3.1. Characteristics of Included Guidelines

WHO recommendations served as the reference standard in this study. They were compared with Zambian guidelines used in perinatal healthcare. Some guidelines were developed by the Zambian Ministry of Health, while others were issued by professional associations. In addition, broader clinical guidelines covering antenatal, intrapartum, and postnatal care were also included (Table 1). Table 3 below summarizes the number and percentage of WHO recommendations according to their level of alignment with Zambian guidelines.

#### 3.1.1. Recommendations for Antenatal Care

A total of 14 WHO recommendations on antenatal care were assessed. Of these, 12 were aligned and 2 were partly aligned. None were classified as modified or does not aligned. The recommendations that aligned included at least eight scheduled antenatal care contacts; daily supplementation with 30–60 mg of iron and 0.4 mg of folic acid to prevent maternal anemia, low birth weight, puerperal sepsis, and preterm birth; and the recommendation that every pregnant woman should carry her own maternity case notes.

However, two WHO recommendations (*n* = 2) were classified as partially aligned. The WHO recommendation states that all pregnant women are advised to receive intermittent preventive treatment with sulfadoxine-pyrimethamine (IPTp-SP) in malaria-endemic areas. Although Zambia follows the timing and monthly interval, the guidelines require pregnant women living with HIV to receive cotrimoxazole and therefore not receive IPTp-SP. Furthermore, the WHO recommendations specify a seven-day antibiotic regimen for asymptomatic bacteriuria; the included Zambian guidelines recommend antibiotics but do not specify the seven-day regimen. It is also noteworthy that despite the Zambian guidelines recommending eight scheduled contacts, only 4.7% (ZDHS 2024, Table 9.2, p. 156) of pregnant women and adolescent girls attended all eight scheduled antenatal contacts, while only 72.4% (ZDHS 2024, Table 9.4, p. 163) had the tetanus toxoid vaccination (Table 4) [13].

#### 3.1.2. Recommendations for Intrapartum Care

Of the 15 WHO recommendations *(n* = 15) on intrapartum care, 13 were aligned with Zambia’s guidelines. These include respectful maternity care that upholds women’s dignity; prophylactic uterotonics such as oxytocin 10 International Units to prevent postpartum hemorrhage for all births in low-resource settings; other uterotonics may be used, such as oral misoprostol 600 μg, if suitable; ergometrine, and performing vaginal examination at four-hour intervals (Table 4).

Two recommendations were classified as only partially aligned with WHO recommendations. These concerned women’s choices regarding pain relief during labour, including epidural analgesia and the definition of the latent and active phases of the first stage of labour. According to WHO, the latent phase is characterized by painful uterine contractions and cervical dilatation of up to 5 cm. It is also noteworthy that some recommendations included in the Zambian guidelines were not fully consistent with WHO recommendations. For example, guidance on informing women about the expected duration of the different phases of labour was presented inconsistently across the reviewed guidelines (Table 4).

#### 3.1.3. Recommendations for Postnatal Care

As shown in Table 4, a total of 22 WHO postnatal care recommendations were included in the review. The majority of the WHO recommendations (*n* = 15) aligned with Zambian guidelines; these included: Regular physiological assessments during the postpartum period are recommended. Comprehensive health education on family planning and task sharing among diverse healthcare providers and cadres. However, the use of a validated tool for postnatal assessment, physical activities aiming for at least 150 min, and the timing of four scheduled postnatal contacts partly aligned. WHO recommends facility care for at least 24 h after birth, followed by contacts at 48–72 h, 7–14 days, and 6 weeks. The reviewed Zambian guidelines differed in their recommended timing. (PNPNC p.M4) indicates contacts within 48 h, at 6–14 days, and at 6 weeks, whereas (MSM p.15) indicates contacts at 6 h, 48 h, 6 days, and 6 weeks. Furthermore, it is noteworthy that, according to the Zambia Demographic and Health Survey 2024, 84.3% of women received postnatal care within the first two days of delivery (ZDHS 2024, Table 9.12, p. 168) [13].

In addition, one of the WHO guidelines (*n* = 1) did not align with the Zambian recommendation. The WHO recommendation indicated that the first bath for term newborns should be delayed by at least 24 h, while Zambian guidelines indicated that the first baby bath should not be done until at least 6 h (Table 4).

#### 3.1.4. Gaps/Challenges

Implementation gaps in maternal and newborn healthcare in Zambia, despite the alignment of national policies with WHO recommendations, are often due to health system limitations, delayed initiation of maternal healthcare services, inadequate maternal knowledge, and sociocultural practices. These systemic and contextual factors hinder the translation of policy into high-quality clinical practice.

## 4. Discussion

The review evaluated the 51 recommendations issued by the WHO on perinatal care and compared them with the Zambian guidelines. These findings are consistent with previously published evidence on perinatal care in Zambia. The comparison demonstrates that in Zambia, WHO recommendations on antenatal, intrapartum and postnatal care are largely aligned. However, their implementation may be constrained by sociocultural factors [50,51,72], workforce shortages [43,44,45,46] broader health system limitations [42,70,73,74], and equipment shortages [36,56].

Furthermore, this review examined the alignment between the recommendations and the feasibility of their implementation by assessing gaps and challenges. A substantial proportion of Zambia’s perinatal health guidelines align with WHO recommendations for antenatal, intrapartum, and postnatal care. However, important gaps remain in the delivery of care. This further highlights the critical difference between guideline adoption and the swift translation of guidelines into practice. For instance, findings from the Zambian Demographic and Health Survey indicated that only 4.7% of pregnant women and adolescent girls had eight ANC contacts, while 84.3% of mothers had postnatal contact within the first two days of delivery [13]. These findings underscore the WHO recommendations and Zambia guidelines on antenatal care and postnatal care. This pattern has been observed across various low- and middle-income countries where adoption occurs, but a significant gap remains in translation.

Similar findings have been reported in other low- and middle-income countries (LMICs), where national maternal and newborn health policies have increasingly been aligned with WHO recommendations, but challenges remain in translating policy into practice. In Ethiopia, effective coverage of antenatal care continues to be influenced by health system, socioeconomic, and service-related barriers despite the adoption of evidence-based maternal health policies [75]. Similarly, studies from Tanzania have shown that implementation of updated antenatal care guidelines is often constrained by workforce shortages, training needs, and limited resources available to nurses and midwives [76,77]. In Uganda, gaps in the quality and continuity of perinatal care have been associated with adverse perinatal outcomes despite the existence of national maternal and newborn care guidelines [78]. These findings mirror the challenges identified in Zambia and suggest that alignment of national guidelines with WHO recommendations is a necessary but insufficient condition for improving maternal and newborn health outcomes. Effective implementation requires sustained investment in health-system capacity, workforce development, and continuity of care.

The adoption of internationally recognized maternal and newborn health standards represents an important step toward improving health outcomes especially in settings that experience high maternal and neonatal mortality [79,80,81]. It is also noteworthy that, notwithstanding the adoption of WHO recommendations, the analysis revealed gaps in ensuring their effective implementation in improving health outcomes. The driving forces often lie within health-system constraints, such as shortages of healthcare personnel, or broader constraints, such as inadequate maternal knowledge. There are thematic guidelines consistent with previous evidence illustrating how weak systems undermine the impact of a well-designed recommendation [81].

In Zambia, recommendations are distributed into multiple guideline documents [19,20,21,22,23]. This suggests that individual healthcare professionals providing perinatal healthcare should read the guidelines to deliver evidence-based practice, considering that a single recommendation and available resources could reduce the burden of guideline overload and improve outcomes, thereby reducing inequities through the provision of standardized care [80,81]. Furthermore, the cultural and social perceptions of the population regarding health issues may explain why some recommendations are not fully implemented. For example, perinatal mental health is a topic with stigma. Although the reviewed Zambian guidance [19] includes structured questions about depressive symptoms, it does not specify a validated screening tool or explicitly include postpartum anxiety. This underscores the aim of comprehensive care. Evidence from Zambia supports strengthening the identification and management of perinatal mental health conditions [63,64].

The success of implementing WHO recommendations relies heavily on context-sensitive adaptation, particularly in resource-constrained settings. In Zambia, greater emphasis lies on the participatory process involving various stakeholders, which is critical for adaptation. The findings further indicate that improving perinatal outcomes requires policies focused on strengthening health system capacity. The integration of WHO recommendations with the Zambian health system quality framework can foster greater uptake and sustained quality in the delivery of perinatal care.

## 5. Conclusions

Most Zambian guidelines align with WHO recommendations, while ten were classified as partially aligned and one did not align. However, gaps lie in the transition of recommendations to clinical practice within Zambian public health services. Addressing these implementation gaps is critical for comprehensive perinatal health care. Furthermore, the Ministry of Health may consider consolidating and updating the existing maternal and newborn health guidelines into a single national perinatal care guideline to support standardized implementation and reduce guideline fragmentation across healthcare settings. Consolidating Zambia’s recommendations, improving health systems, and filling gaps in recommendations will encourage the delivery of standardized, high-quality perinatal care to enhance maternal and child health and well-being. These actions may contribute to achieving Sustainable Development Goal 3 by reducing preventable maternal and newborn morbidity and mortality.

## 6. Study Strengths and Limitations

This review was conducted using internationally recognized methods for evidence synthesis. This ensured that the findings were transparent and reliable. The approach also assessed the areas of non-alignment, gaps, and challenges associated with each recommendation. Only officially published guidelines were intended for use in Zambia. However, the review had some limitations, as the searches were mainly conducted for published articles online. This meant there could be guidelines that used as national wide for perinatal care but were not published online. Additionally, while this current article focused on perinatal care, most guidelines only extend up to the 6th week of postpartum, meaning there is a lack of evidence between the six weeks up to one year after childbirth. Furthermore, although two reviewers reached a consensus agreement, the risks remain of subjective interpretation because the mapping process involved interpretive assessment.

## Figures and Tables

**Table 1:** National documents used for mapping.

Document Title	Year	Organisation	Scope
Pregnancy, Childbirth, Postpartum, and Newborn care: A guide for essential practice in Zambia (PNPNC) [19]	2016	MoH	Antenatal care, intrapartum, postnatal care and newborn care
Zambia Consolidation Guidelines for Treatment and Prevention of HIV infection (ZCG) [20]	2022	MoH	Prevention of mother- to- child transmission during pregnancy, childbirth and breastfeeding
ANC Guidelines for a positive pregnancy Experience (ANCg) [21]	2018	MoH	Antenatal care
TASKPEN clinical guidelines [22]	2022	CIRDZ, UTH, UNZAsom, LAMU	Diagnosis and management of diabetes in pregnancy
Midwifery Care Standards of Care (MSM) [23]	2024	NMCZ	Continuum of maternal and newborn care in midwifery

Abbreviations: CIRDZ: Centre for Infectious Disease Research in Zambia; LAMU: Lusaka; Apex Medical University; MoH: Ministry of Health; NMCZ: Nursing and Midwifery; Council of Zambia; UNZAsom: University of Zambia School of medicine; UTH: Teaching Hospital.

**Table 2:** Mapping Recommendation.

Classifications	Definition
Aligns	The recommendation is consistent with the WHO recommendation.
Partly aligns	The recommendation reflects some, but not all, components of the WHO recommendation.
Modified	The recommendation retains the overall intent of the WHO recommendation but has been adapted to the Zambian context in its content, scope, or implementation approach.
Does not align	The recommendation differs substantially from or contradicts the WHO recommendation.

**Table 3:** Summary of alignment between WHO recommendations and Zambian guidelines.

Care Domain	WHO Recommendations	Aligns	Partly Aligns	Modified	Do Not Align
	*n*	*n* (%)	*n* (%)	*n* (%)	*n* (%)
Antenatal care	14	12 (85.7)	2 (14.3)	0 (0.0)	0 (0.0)
Intrapartum care	15	13 (86.7)	2 (13.3)	0 (0.0)	0 (0.0)
Postnatal care	22	15 (68.2)	6 (27.3)	0 (0.0)	1 (4.5)
Total	51	40 (78.4)	10 (19.6)	0 (0.0)	1 (2.0)

**Table 4:** Comparison of WHO Recommendations and Zambian guidelines on comprehensive perinatal care.

WHO Recommendations	Zambian Guidelines	Source	Alignment	Challenges
**Antenatal** [9] **Nutritional assessment** Counseling on healthy eating habits and physical activities	Nutritional counseling and physical activities is provided [24,25,26]	MCS p.8, PNPNC p.C13	Aligns	Inadequate maternal knowledge, resulting from the inconsistency in the information given on ideal exercises [26]
Iron 30–60 mg and folic acid 0.4 mg supplement	Supplements iron 30–60 mg and folic acid 0.4 mg are recommended. At least 94% of pregnant women take supplements [13,27]	ANCg p.25	Aligns	Late ANC initiation and adherence issue [28,29]
**Maternal and fetal assessment** Categorization of hyperglycemia as gestational diabetes mellitus or diabetes mellitus	Uses 20 weeks gestation as a benchmark to differentiate gestational diabetes from pre-existing diabetes mellitus	TASKPEN p.42	Aligns	Health system and related limitations: urine and blood testing are often underutilized [30,31,32]
Inquiring about substance and alcohol usage	History inquiring is done on alcohol and tobacco usage if necessary, counselling is done	ANCg p.34–37 PNPNC p.C6,	Aligns	Lack of and inconsistency in screening; inadequate maternal knowledge [33]
Provider-initiated testing and counselling (PITC) for HIV	PITC done at every first ANC visit and follow up at every 3 months through out pregnancy	ANCg p.34	Aligns	Low antenatal attendance, social-cultural practices, and poor health-system practice [31,34,35]
Ultrasound before 24 weeks of Gestation	1st ultrasound at 18–20, the other at 28 weeks	ANCg p.34, ZCG p.31	Aligns	Access challenges to equipment, Low antenatal attendance, and maternal health knowledge and health literacy [36,37]
**Preventive measures** A seven-day antibiotic regimen is advised for all pregnant women diagnosed with asymptomatic bacteriuria (ASB)	Testing of all expecting mothers through routine urinalysis. If diagnosed with ASB, antibiotics are the first line of treatment. However, number of days for the antibiotic regimen is not given	ANCg p.13	Partly Aligns	Health system and related limitations; urine and blood testing is often underutilized [30,31,32,38]
Tetanus toxoid vaccination in consideration of the last dose	Can be administered at any time until the 6th contact, 34–35 weeks. At least 72.4% had the tetanus dose [13]	ANCg p.34–36, PNPNC p.C12	Aligns	Inadequate maternal knowledge [39]
Preventive anthelminthic treatment is recommended after the 1st trimester	A single dose is recommended after the first trimester	ANCg p.19, PNPNC p.F3	Aligns	Low antenatal attendance or late antenatal initiation [30]
All pregnant women are advised to have intermittent preventive treatment with sulphonamides- pyrimethamine (IPTp-SP). The second trimester is when the dosage should begin, and the dose must be administered for at least one month apart.	While most pregnant women are given at least 4 doses starting at the third contact (21–26 weeks). Those Living with HIV can be beneficial compromise when given IPTp-SP It was estimated that at least 70% had recommended doses [13]	ANCg p.34–36	Partly Aligns	Inadequate maternal knowledge [40,41]
**Health systems interventions** Advised that every pregnant mother carry her own case notes	Pregnant women and adolescent girls carry their own case in form of a book or card [39]	ANCg p.8	Aligns	Health system and related limitations: paper-based data storage [42]
Have broadened ranges of tasks shifting among skilled health professions with promoting health-related behaviors for mother and newborn health	Doctors, midwives, and Nurses and other health cadres work hand in hand [42,43]	ANCg p.34–37	Aligns	Health system and its related limitations [43,44]
Delegating the provision of Recommended Nutritional Supplement and IPTp to a broad range of health professionals	IPTp and nutritional supplements are given at the ANC service point. To increase coverage of other health cadres’ delegates [29]	ANCg p.34–37	Aligns	Health system and related limitations [45,46]
Eight scheduled visits	Eight scheduled contacts for pregnant women and adolescent girls are recommended. Current estimates show 82% had at least four. Furthermore, only 4.7% had at least 8 recommended ANC visits [13]	ANCg p.34–37	Aligns	Low ANC attendance and Late ANC initiation [47,48,49]
**Intrapartum [17]**				
Respectful maternity care that upholds women’s dignity	During labour and delivery women and adolescent girls should be given care that maintains their dignity and utmost respect	MSM p.9, PNPNC p.C2	Aligns	Health system and limitations Health care providers believe that disrespectful care is the only way to meet clinical objectives and achieve better outcome [50,51,52]
Effective communication	Timely and effective communication is encouraged for laboring women	MSM p.3, PNPNC p. C2	Aligns	Health system and limitations: Shortage of healthcare professionals [50,51,52]
**First stage of Labor** Using the definition of Latent and active phase (5 cm)	Definition of latent stage and the active stage differ Zambia guideline indicate 4 cm for active labour	PNPNC p.D3	Partly aligns	None
Information on the duration of the phases should be provided to the woman	Women are to be informed about the progress of labor	MSM p.12, PNPNC p.D6	Aligns	Shortage of healthcare professionals [50,51,52,53]
Auscultation can be done using a Doppler ultrasound device or Pinard fetal stethoscope	Depending on the context, Doppler ultrasound devices or a pinard fetal stethoscope is used	MSM p.10	Aligns	None
Performing a four-hour interval of vagina examination	Vaginal examination to be performed at a four-hour interval	MSM p.12, PNPNC p.D8	Aligns	None
Taking into consideration pregnant women’s choices about pain relief, epidural analgesia is recommended; additionally, opioids such as fentanyl, diamorphine, and pethidine are options that are recommended. Relaxation and manual techniques can be used	Pain-relief methods are recommended, but the guideline does not clearly emphasize the woman’s choice. Pharmacological agents may be used below 6 cm cervical dilatation, while non-pharmacological methods may be used during contractions	MSM p.12	Partly aligns	Health system and limitation: inadequate maternal knowledge [54].
Women who are low risk can take oral fluids and food	Women have the freedom to eat or drink if at low risk	MSM p.12, PNPNC p.D6	Aligns	None
The second stage is the period between full cervical dilatation and the baby’s birth	Period at 10 cm, bulging thin, head visible	PNPNC p. D10	Aligns	None
Women can adopt the birth position that they choose, including an upright position	Women can choose the position they want to deliver their babies, but this is not fully implemented	MSM p.12, PNPNC p. D10	Aligns	None
Woman’s own urge to push should be encouraged and supported	women are encouraged to push on their own urge	PNPNC p. D10	Aligns	None
Available options and women’s preference can be considered when preventing perineal tears and trauma; this could include warm compress and hand on	Hand-on techniques is usually performed.	PNPNC p. D11	Aligns	None
Prophylactic uterotonics such as oxytocin, oral misoprostol, ergometrine	Prophylactic uterotonics are used for routine [54]	PNPNC p.D11	Aligns	Health system and limitation [55]
Cord clamping 1 min after birth helps with nutrition and health outcomes	Between 1 min and 3 min, except in situations when the newborn may need resuscitation [55]	MSM p.12 PNPNC p.D11	Aligns	Health system and limitation
Cord controlled traction with the help of a skilled birth attendant	Controlled traction is recommended to deliver the afterbirth by the skilled health professional	PNPNC p.D12	Aligns	None
**Postnatal [18]**				
Postpartum women should undergo regular physiological assessments within the first 24 h after birth. All vital signs and urine void should be recorded within 6 h. Subsequent assessment should continue beyond this period, focusing on general well-being	Postnatal assessments are recommended on regular physiological during the first hour, assessments be conducted at 15-min intervals. For two hours, every 30 min; for three hours, hourly	MSM p.13 PNPNC p.D19	Aligns	Health system and limitation: shortage of healthcare professionals [56,57]
In high HIV-burden settings, postpartum HIV testing is needed for women who missed antenatal or third-trimester testing	Women who missed testing or previously tested negative are retested [57]	PNPNC p.E5 ZCG p.31	Aligns	Health system and limitation [58]
Interventions for common physiological signs and symptoms: Offer ice packs or cold pads after childbirth to relieve acute perineal pain, depending on the woman’s preference and availability	General management of postpartum discomforts as being included	MSM p.16	Partly aligns	Health system and limitation
Oral analgesia can be used to reduce pain in the perineum during postnatal. For uterine cramping pain, oral non-steroidal anti-inflammatory drugs can be used based on the woman’s preferences	Systemic analgesics are the first-line treatment recommended	PNPNC p.E7	Aligns	None
Support women with responsive breast-feeding, appropriate positioning, milk expression, and, if preferred, warm or cold compresses for postpartum breast engorgement	Women are encouraged to breast-feed responsively to prevent postpartum breast engorgement	PNPNC p.K3, J9	Aligns	None
**Preventive measures** To prevent postpartum mastitis, women should be counselled on responsive breastfeeding, baby positioning, hand expression of breast milk, and using warm or cold compresses based on their preferences	Women are encouraged to breastfeed responsively to prevent mastitis.	PNPNC p.K3, J9	Aligns	Social-cultural practices, Health system Limitation [59,60]
Dietary advice should be provided to women to prevent postpartum constipation	Nutritional counselling is included in postnatal care	MSM p.16, PNPNC p.D26	Aligns	Social-cultural practices, Health system limitation [61,62]
**Mental health interventions** Use validated tools to screen for postpartum depression and anxiety and provide diagnosis and management for women who screens positive	Structured questions are used to assess symptoms, and women are referred for further management according to their responses. However, the guideline does not specify a validated tool or explicitly include postpartum anxiety	PNPNC p.E7	Partly aligns	Health system limitation, social-cultural practices [63,64]
Psycho-social and psychological interventions should be advocated	Encourages family and community support for pregnant [54]	PNPNC p.I2	Aligns	Health system and limitation, social-cultural practices [63,64]
**Physical activities** Postpartum women without contraindications should aim for at least 150 min of mixed physical activity	Supports the gradual resumption of physical activity but does not specify the 150 min of moderate -intensity	MSM p.16	Partly aligns	None
**Contraceptive** Comprehensive information on contraceptives should be given	Information on contraceptives is provided	MSM p.16, PNPNC p.D27	Aligns	Health system and limitation, social-cultural practices [65,66,67]
**Newborn Assessment** Newborns should be assessed for danger signs, and parents and families should be encouraged to seek health care services early when they recognize these signs	Health education is taught at discharge and follow-up postnatal schedule	PNPNC p.D28	Aligns	Health system limitation, social-cultural practices, maternal knowledge [68,69]
Abnormalities of the eyes and ears should be assessed, and screening for hyperbilirubinemia should be performed using a transcutaneous bilirubinometer	Recommendation of general newborn examination and jaundice assessment, but does not require transcutaneous bilirubin before screening before discharge	PNPNC p.J6 MSM p.13	Partly aligns	Health system and limitation [68]
**Preventive Measures** The first bath for term newborns should be delayed by at least 24 h	The guideline states that the first baby bath should not be done until at least 6 h	PNPNC p. J10	Does not align	Social and cultural practices [69,70,71]
The umbilical cord should be kept dry and clean	Mothers are taught and encouraged to keep the umbilical cord dry and clean before discharge. Follow-up check is done	PNPNC p. K10	Aligns	Social and cultural practices [71,72]
To prevent sudden infant death syndrome (SIDS) and sudden unexpected death in infancy, the supine position is Recommended	The guideline recommends not only supine but also lying the baby on the back or sidewise	PNPNC p. K10	Partly aligns	Social and cultural practices [69,70,71,72]
Postnatal care is a minimum of 4. These are as follows: at birth in the health facility for 24 h, between 48 and 72 h, 7 and 14 days, and 6 weeks after. Women after vaginal birth can stay for 24 h if healthy	Postnatal care is also scheduled at a minimum of 4 contacts. However, the two guidelines are inconsistent. Postnatal contacts are scheduled in both guidelines, but their recommended timing differs. PNPNC (p. M6) indicates contacts within 48 h, at 6–14 days, and at 6 weeks, whereas MSM (p. 15) indicates contacts at 6 h, 48 h, 6 days, and 6 weeks. According to the 2024 ZDHS, 84.3% of women received postnatal care within two days after delivery [13]	MSM p.15 PNPNC p.M6	Partly aligns	Health system and limitations [70,71]
Women should be assessed for the following criteria for discharge. Physical well-being, emotional well-being, and her being confident about self-care	Assessment of mother for discharge is done	MSM p.16	Aligns	Health system and limitation: Shortage of healthcare professional [70]
Women should be provided with information, educational interventions, and counseling.	Health Education is provided	MSM p.16	Aligns	Shortage of healthcare professionals, Health system, and limitation [70]
Health professionals and Community health workers can do home visits. However, where not feasible; postnatal outpatient visits are feasible	Postnatal is provided through maternal health services clinic	MSM p.15	Aligns	Health system and limitation: shortage of healthcare professionals [70]
Task sharing among diverse health workers and cadres	Recommends tasks coordination among health care providers, community volunteers, safe -motherhood groups	PNPNC p.I2	Aligns	Health system and limitation: shortage of healthcare professionals [44,46,70]
It is recommended to use both home-based and hospital-based records	Hospital-based and home-based records are recommended	PNPNC p. E3	Aligns	Health system and limitation [42,73]

Abbreviations: ANCg: ANC guidelines [21]; MCS: Midwifery Care Standards [23]; PNPNC: A guide for essential practice [19]; TASKPEN guideline [22]; ZCG Zambia Consolidation [20].

## Data Availability

No new data were created or analyzed in this study. Data sharing is not applicable to this article.

## Abbreviations

The following abbreviations are used in this manuscript:

ANC	Antenatal Care
ANCg	ANC Guidelines for a positive pregnancy Experience
CIRDZ	Centre for Infectious Disease Research in Zambia
IPTp-SP	Intermittent preventive treatment with sulfonamides-pyrimethamine
LAMU	Lusaka Apex Medical University
LMIC	Low- and middle-income countries
MCS	Midwifery Care standards
NMCZ	Nursing and Midwifery council of Zambia
PITC	Provider-initiated testing and counseling
PNPNC	Pregnancy, Childbirth, Postpartum, and Newborn care: A guide for essential practice
PRISMA	Preferred Reporting Items for Systematic Reviews and Meta-Analyses
SDG	Sustainable Development Goals
WHO	World Health Organisation
ZCS	Zambia consolidation guidelines for treatment and prevention of HIV infection
ZDHS	Zambia Demographic Health Survey
